# A bootstrapping approach for generating an inverse distance weight matrix when multiple observations have an identical location in large health surveys

**DOI:** 10.1186/s12942-019-0189-5

**Published:** 2019-11-25

**Authors:** Sung Wook Kim, Felix Achana, Stavros Petrou

**Affiliations:** 1 Warwick Clinical Trials Unit, Division of Health Sciences, Warwick Medical School, University of Warwick, Coventry, CV4 7AL UK; 2 Nuffield Department of Primary Care Health Sciences, University of Oxford, Radcliffe Observatory Quarter, Woodstock Road, Oxford, OX2 6GG UK

**Keywords:** Spatial weight matrix, Bootstrapping, Large surveys, Inverse distance, Spatial lag

## Abstract

Spatial weight matrices play a key role in econometrics to capture spatial effects. However, these constructs are prone to clustering and can be challenging to analyse in common statistical packages such as STATA. Multiple observations of survey participants in the same location (or cluster) have traditionally not been dealt with appropriately by statistical packages. It is common that participants are assigned Geographic Information System (GIS) data at a regional or district level rather than at a small area level. For example, the Demographic Health Survey (DHS) generates GIS data at a cluster level, such as a regional or district level, rather than providing coordinates for each participant. Moreover, current statistical packages are not suitable for estimating large matrices such as 20,000 × 20,000 (reflective of data within large health surveys) since the statistical package limits the N to a smaller number. In addition, in many cases, GIS information is offered at an aggregated level of geographical areas. To alleviate this problem, this paper proposes a bootstrap approach that generates an inverse distance spatial weight matrix for application in econometric analyses of health survey data. The new approach is illustrated using DHS data on uptake of HIV testing in low and middle income countries.

## Introduction

Spatial weight matrices play an important role in econometrics to capture spatial effects [1]. These matrices are used to generate spatial lag variables and spatial error models [2]. Unfortunately, however, Geographic Information System (GIS) data are commonly provided at an aggregated geographic level in many national and international health surveys. In other words, participants are generally assigned a GIS location at a regional or district rather than small area level.

It is a common procedure that data surveyors attempt to aggregate collected data at a higher level in order to conceal the identity of survey participants. In terms of spatial data, one way of hiding the identity of participants is to aggregate individual-level to a higher level such as region or cluster [3]. The Demographic and Health Survey (DHS) data uses the aggregation approach to protect respondents’ confidentiality. As another example, UNICEF’s Multiple Indicator Cluster Survey (MICS) collects cluster level data but only reports the regional level, which is a higher level of data [3]. In addition to these examples, the Centres for Disease Control and Protection (CDC) and US Census Bureau also apply an aggregation approach in their health surveys [3].

Given this issue, the following analytical challenges can arise. Generating spatial weight matrices based on distance using multiple observations of survey participants in the same area, such as households located in an identical location (or cluster), is not currently possible. This is mainly because multiple observations in the same location have identical information regarding longitude and latitude, so the distances between the observations become zero. Spatial regression assumes that every observation has unique location information. As such, a spatial weight matrix based on distance such as k-nearest neighbour or inverse distance cannot be generated in analyses using these data.

Moreover, it may not be possible to generate a spatial weight matrix since commonly used statistical packages have limitations in estimating a large size matrix. For example, the STATA statistical package limits the number of N to 11,000. Consequently, statistical packages that calculate spatial weight matrices such as ‘SPMAT’ [4] and ‘spwmatrix’ [5] do not function for datasets that exceed N = 11,000. Likewise, the limit of vectors that can be used within the R package is 2,147,483,647; however, this is not suitable for a 4 GB memory computer and inevitably requires additional memory [6]. One alternative is to use a special matrix language such as ‘Mata’ in STATA because Mata has no limits in calculating the matrix [7]; however, it can be burdensome for researchers to learn another statistical language. In practice, analyses of many national and international health survey datasets face both of these methodological challenges, and the existing literatures does not suggest a way of alleviating these challenges [8–10].

This study therefore presents a novel bootstrap-based method approach for generating an inverse distance weight matrix when multiple observations have an identical location in large health surveys.

## Methods

### Spatial weight matrix

A spatial weight matrix is used to represent the spatial structure within a dataset. The spatial weight matrix, **W**, is an n x n non negative matrix that has an element of $W_{ij}$, which is the weight at each location i, j. There are a number of approaches to generate a spatial weight matrix [10]. Amongst them, the spatial inverse distance weight matrix is a popular method as it is relatively simple to calculate the weights [8]. The spatial inverse distance weight matrix can be expressed as

1 $$ W_{ij} = \left\{ {\begin{array}{*{20}c} {\frac{1}{{d_{ij}^{\alpha } }}   \quad if\; i \ne j} \\ {0      \quad if\; i = j} \\ \end{array} } \right.. $$

In general, a spatial lag model is expressed as:

2 $$ y = \rho Wy + X\beta + \varepsilon $$

where ρ is a coefficient for a spatial lag variable *y* is a *n *× 1 vector of the dependent variable, *W* is a *n *× *n* spatial weights matrix, *e* is a vector of error terms, and β is a vector of regression coefficients [10]. The concepts of Moran’s I and the bootstrap method are explained in Appendix.

### The reliability of simulation

The reliability of a simulation can be confirmed by estimating the following concepts: coverage probability and mean squared error (MSE) [11, 12]. Coverage probability represents the probability that the confidence interval contains the true value of the variable of interest. In general, approximately 95% probability of coverage is said to be reliable [13]. The accuracy of the simulation can also be checked using MSE statistics [14]:

$$ MSE = 1/N\mathop \sum \limits_{j} ( \hat{\beta }_{j} - \beta )^{2} $$

where N is the total number of observations and $\beta$ is a true value of the parameter. $\hat{\beta }$ is the value of the bootstrap simulation. It is desirable to have a MSE value close to zero [13].

### Basic idea of the model

This study focuses on the following comparison. An inverse distance weight matrix was generated without random sampling using original DHS data. Distance was defined as Euclidean distance [15]. To avoid the technical errors derived from the insufficient memory, an inverse distance weight matrix was generated with Mata language using STATA [7]. Furthermore, another inverse distance weight matrix based on random sampling was generated in order to compare the result with the matrix generated using the Mata language. To do so, 10,000 bootstraps were performed, selecting one observation from one cluster; that is, a total of 850 observations were used to generate the spatial weight matrix using the bootstrap method within the SPMAT package [4]. A bootstrap method was carried out with ‘bsample’ and ‘simulate’ commands in STATA [16]. This random sampling can avoid the problem that the denominator in Eq. (1) becomes zero as a result of multiple observations being given identical coordinates. Regardless of the number of iterations, this matrix will be constant because a random sample drawn from each of the clusters offers identical distance, given the constant distance between clusters. A spatial probit model [17, 18] was also considered as the outcome variable in our applied example is a binary variable.

Based on the literature about the association between spatial access to HIV care [20–22] and education [20] and income [19–22], the spatial lag model used in this study is as follows.

$$ HIV\; testing = \rho W*HIV\; testing + \beta_{1} *{\text{income }} + \beta_{2} *{\text{education}} + \varepsilon . $$

### Sensitivity analysis

An alternative dependent variable (visiting any type of health services over the last 12 months) was also selected because it showed a higher value of Moran’s I (0.009 for women and 0.01 for men) than that for the variable of ‘HIV testing’ in the study dataset. Based on the existing literature [20, 23, 24], a model of using ‘visiting health services’ as a dependent variable, and wealth and education as independent variables was also considered.

### Data

DHS data for Malawi was used for this study. This survey provides nationally representative data for several developing countries with respect to socioeconomic status such as wealth, as well as clinical information such as mode of delivery and HIV testing [25]. The DHS collects GIS data at a cluster level rather than providing coordinates for each observation of a participant. As an example, DHS Malawi 2015–2016 offers only 850 cluster level GIS values for approximately 24,000 participants. The focus of this study is on HIV test uptake, which is defined as ‘ever tested for HIV’. This data was obtained from women and men age 15–49 years and covers the lifetime of the respondent [26].

## Results

A descriptive table of data used in this study is provided in Appendix. The analysed dataset includes 7289 women and 17,273 men. Both samples were drawn from 850 clusters.

### Moran’s I

Table 1 shows results for Moran’s I statistic. The statistic for Moran’s I is close to zero, suggesting that spatial autocorrelation in this study was weak. Nevertheless, the p-values for the Moran’s I statistic are significant for both women and men (p < 0.001). The bootstrap simulation result shows a small difference from the original result. For women, the Moran’s I statistics based on the original data and 10,000 bootstrap iterations were 0.004 and 0.002, respectively. Similarly, Moran’s I statistics for men were 0.003 and 0.002, respectively. The sign for the coefficients for the simulated results is identical to that for the original data and the result provides a small bias. These results suggest that the bootstrapping simulation offers close results to those based on the original data despite the weak spatial autocorrelation.

**Table 1:** Moran’s I statistics

	Women				Men		
	Moran’s I	Standard deviation	P-value		Moran’s I	Standard deviation	P-value
Original data	0.004	0.001	0.000	Original	0.003	0.0003	0.000
10,000 iteration	0.002	0.005	0.267	10,000 iteration	0.002	0.006	0.260

### Regression results

Table 2 presents the regression results using the original data and using bootstrap simulations. The reliability of the bootstrapped results is checked using coverage probabilities and mean squared errors [11]. For women and men, as an example, the coverage probabilities following 5000 iterations of the wealth variable were 95.3% and 95.1%, respectively. In the same manner, the coverage probabilities for the wealth variable following 10,000 bootstrap simulations were 95.0% and to 95.6%, respectively.

**Table 2:** OLS regression (HIV testing)

	Coef^b^	SE	CI (lower)	CI (higher)	Coverage probability (%)	MSE
Women						
Original data^a^						
Spatial lag	1.159	0.226	0.716	1.603		
Wealth	0.003	0.003	− 0.003	0.010		
Education	0.008	0.007	− 0.007	0.022		
5000 simulation						
Wealth	0.008	0.009	− 0.010	0.026	94.5	0.0001
Education	0.011	0.018	− 0.025	0.046	97.8	0.0003
10,000 simulation						
Wealth	0.008	0.009	− 0.010	0.026	95.0	0.0001
Education	0.011	0.018	− 0.024	0.046	97.9	0.0003
Men						
Original data^a^						
Spatial lag	1.337	0.171	1.001	1.672		
Wealth	− 0.011	0.002	− 0.016	− 0.007		
Education	0.018	0.005	0.009	0.027		
_cons	− 0.270	0.144	− 0.552	0.013		
5000 simulation						
Wealth	− 0.010	0.009	− 0.029	0.009	95.1	0.0003
Education	0.019	0.019	− 0.019	0.056	96.6	0.0005
10,000 simulation						
Wealth	− 0.010	0.009	− 0.028	0.008	95.6	0.0001
Education	0.019	0.019	− 0.019	0.056	97.0	0.0004

*MSE* mean squared error

^a^Row normalised

^b^This was estimated using spmat and spreg package in Stata

MSE values obtained by bootstrapping were close to zero. The MSEs following both 5000 and 10,000 iterations for men were 0.0001 (wealth) and 0.0004 (education), respectively. Likewise, the MSEs for the wealth and education variables for women were 0.0001 and 0.0003, respectively. One recommended approach for using the confidence interval is to check the reliability of simulation results [12]. Although it is not possible to accurately estimate this parameter as the confidence interval changes from a negative to a positive sign, the values of the regression coefficients from the original data fall into the bootstrapped confidence interval of the simulated data.

Table 3 presents the regression results using a spatial probit model. It can be seen that there is no difference between 5000 iterations and 10,000 iterations in terms of the magnitude of coefficients. The coefficient values are contained in the bootstrap confidence intervals (− 0.036 to 0.104 for the wealth variable following 10,000 iterations; and − 0.095 to 0.198 for the education variable following 10,000 iterations). Moreover, the coverage probabilities are close to 95%. For men, the independent variables show a similar pattern. The coefficient values are close to the true values (− 0.048 vs − 0.040 for wealth; 0.079 vs 0.087 for education) and contained in the bootstrap confidence intervals. Again, the coverage probability varies from 94.7 to 96.5%. To sum up, the simulation results are predictive of true values generated from the original data.

**Table 3:** Spatial probit (HIV testing)

	Coef	SE	CI (lower)	CI (higher)		
Women						
splag	4.371	0.865	2.676	6.067		
Wealth	0.013	0.013	− 0.013	0.040		
Education	0.033	0.029	− 0.023	0.090		

	Coef	SE	Boot CI (lower)	Boot CI (higher)	Coverage probability (%)	MSE
5000 simulation						
Wealth	0.035	0.035345	− 0.035	0.104	94.1	0.002
Education	0.051	0.073127	− 0.092	0.194	97.6	0.006
10,000 simulation						
Wealth	0.034	0.035677	− 0.036	0.104	94.4	0.002
Education	0.051	0.074628	− 0.095	0.198	97.3	0.006

	Coef	SE	CI (lower)	CI (higher)	Coverage probability (%)	MSE
Men						
splag	5.506	0.709	4.117	6.895		
Wealth	− 0.048	0.009	− 0.066	0.030		
Education	0.079	0.020	0.040	0.118		
_cons	− 3.570	0.597	− 4.741	− 2.400		
5000 simulation						
Wealth	− 0.040	0.040929	− 0.120	0.040	94.8	0.002
Education	0.086	0.085585	− 0.082	0.254	96.4	0.007
10,000 simulation						
Wealth	− 0.040	0.041274	− 0.121	0.041	94.7	0.002
Education	0.087	0.084701	− 0.079	0.253	96.5	0.007

### Sensitivity analysis

A sensitivity analysis was performed using another dependent variable (visiting health services) that had a higher Moran’s I values, namely 0.009 for women and 0.01 for men. The simulated results are similar to the estimated values of the coefficients of the regression. The coverage probabilities were 95.4% and 96.6% for wealth and education, respectively. In Table 4, for men and following 10,000 iterations, the values (− 0.012 for wealth and 0.019 for education) were also contained within the bootstrap confidence intervals (− 0.038 to 0.011 for wealth and − 0.035 to 0.072 for education). Again, in Table 5, the values (− 0.031 for wealth and 0.053 for education) fall into the bootstrap confidence intervals. The MSEs were close to zero. In brief, the results of this sensitivity analysis were consistent with the simulated results that used HIV test uptake as the dependent variable.

**Table 4:** Sensitivity analysis—OLS (health service use)

	Coef	SE	CI (lower)	CI (higher)		
Women						
Original data^a^						
Spatial lag	1.645	0.159	1.333	1.956		
Wealth	− 0.012	0.004	− 0.020	− 0.003		
Education	0.029	0.009	0.011	0.048		
Constant	− 0.382	0.094	− 0.566	− 0.198		

	Coef	SE	Boot CI (lower)	Boot CI (higher)	Coverage probability (%)	MSE
5000 simulation						
Wealth	− 0.007	0.012	− 0.030	0.017	95.4	0.000169
Education	0.024	0.025	− 0.025	0.074	96.5	0.000659
10,000 simulation						
Wealth	− 0.007	0.012	− 0.030	0.016	95.4	0.000166
Education	0.025	0.025	− 0.024	0.075	96.6	0.000651

	Coef	SE	CI (lower)	CI (higher)		
Men						
Original data^a^						
Spatial lag	− 0.053	0.045	− 0.142	0.036		
Wealth	− 0.012	0.003	− 0.018	− 0.006		
Education	0.019	0.006	0.007	0.032		
Constant	0.712	0.052	0.611	0.813		

	Coef	SE	Boot CI (lower)	Boot CI (higher)	Coverage probability (%)	MSE
5000 simulation						
Wealth	− 0.014	0.013	− 0.039	0.011	95.3	0.000165
Education	0.019	0.028	− 0.036	0.073	95.2	0.000773
10,000 simulation						
Wealth	− 0.014	0.013	− 0.038	0.011	95.4	0.000161
Education	0.018	0.027	− 0.035	0.072	95.8	0.000752

^a^Row normalised

**Table 5:** Sensitivity analysis—spatial probit model (health service use)

	Coef	SE	CI (lower)	CI (higher)		
Women						
Original data^a^						
splag	4.310	0.420	3.486	5.134		
Wealth	− 0.030	0.011	− 0.052	− 0.008		
Education	0.076	0.024	0.028	0.124		
_cons	− 2.319	0.248	− 2.805	− 1.832		

	Coef	SE	Boot CI (lower)	Boot CI (higher)	Coverage probability (%)	MSE
5000 simulation						
Wealth	− 0.012	0.029	− 0.070	0.045	94.6	0.001
Education	0.068	0.065	− 0.060	0.195	97.0	0.004
10,000 simulation						
Wealth	− 0.012	0.030	− 0.071	0.047	94.4	0.001
Education	0.066	0.065	− 0.061	0.193	96.8	0.004

	Coef	SE	CI (lower)	CI (higher)		
Men						
Original data^a^						
splag	5.419	0.291	4.848	5.990		
Wealth	− 0.031	0.008	− 0.046	− 0.016		
Education	0.053	0.017	0.020	0.086		
_cons	− 3.050	0.187	− 3.417	− 2.683		

	Coef	SE	Boot CI (lower)	Boot CI (higher)	Coverage probability (%)	MSE
5000 simulation						
Wealth	− 0.032	0.033	− 0.097	0.032	96.2	0.001
Education	0.057	0.073	− 0.086	0.199	95.7	0.005
10,000 simulation						
Wealth	− 0.032	0.033	− 0.096	0.032	96.3	0.001
Education	0.057	0.074	− 0.088	0.201	95.7	0.005

^a^Row normalised

## Discussion

This study applies a bootstrap method to generate an inverse distance weight matrix in the context of a large health survey with multiple observations in identical geographical locations. A number of global health surveys use the aggregation approach to protect participants’ identity, so this prohibits researchers from generating distance based spatial weight matrices. This paper attempts to resolve this problem by introducing a bootstrapping method in generating inverse distance spatial weight matrices. Spatial regression using a matrix programming language, Mata, was carried out and the result was compared with the result of spatial regression based on bootstrapping. The results following use of the bootstrap were consistent with the results that used the original data, and coverage probabilities support the bootstrap results provided in this study.

A few limitations need to be noted. Firstly, it was not possible to identify a variable of higher Moran’s I value. It is possible that due to the small Moran’s I value, the spatial lag variable does not sufficiently capture the spatial effect. Consequently, because of the small spatial effect captured by the spatial lag variable, the coefficients for the independent variables will not vary considerably. However, the sensitivity analyses generated consistent results with those using HIV test uptake as the dependent variable even when Moran’s I values increased by ten times for men and two times for women. Secondly, the suggested approach was applied only to a spatial lag model with a binary variable. It is not certain whether consistent results can be obtained for multiple choice models such as the ordered choice model. Despite these limitations, the advantage of using the bootstrap method approach for generating an inverse distance weight matrix is that it is able to simplify the calculation of the spatial weight matrix regardless of the size of a matrix.

In conclusion, this study suggests a simplified approach to generating inverse distance weight matrices for spatial analyses. This methodological approach is likely to be of practical value when big data issues or duplicated GIS information arise.

## Data Availability

Data can be obtained from the DHS website.

## Appendix

- Moran’s I

Moran’s I is a widely used measure to detect spatial autocorrelation. This index ranges from − 1 to + 1. A negative outcome means that there is negative spatial autocorrelation; likewise, a positive outcome means that there is positive spatial autocorrelation.

$$ {\text{I}} = \frac{{N\mathop \sum \nolimits_{i = 1}^{n} \mathop \sum \nolimits_{j = 1}^{n} W_{ij} \left( {X_{i} - \bar{X}} \right)\left( {X_{j} - \bar{X}} \right)}}{{\mathop \sum \nolimits_{i = 1}^{n} \mathop \sum \nolimits_{j = 1}^{n} W_{ij} \left( {X_{j} - \bar{X}} \right)^{2} }} $$

where N is the total number of observations, $\bar{X}$ is the mean of the variable, $X_{i}$ is the value of the variable at the location i, $X_{j}$ is the variable at the location j and W is the spatial weight index.

- Bootstrap method

The bootstrap method was introduced by Efron [27]. $\left\{ {y_{1} , y_{2} , y_{3} , \ldots , y_{n} } \right\}$ denotes the outcome of the random sample to obtain the estimator [28]. This sample is regarded as the population and a random sample of N is drawn from $\left\{ {y_{1} , y_{2} , y_{3} , \ldots , y_{n} } \right\}$. If we draw a random sample from the sample, $\left\{ {y^{\left( t \right)}_{1} , y^{\left( t \right)}_{2} ,y^{\left( t \right)}_{3} , \ldots , y^{\left( t \right)}_{n} } \right\}$ denotes the randomly drawn sample. The M-estimator is used to minimise the sum of functions of the data. $\hat{\theta }^{\left( t \right)}$, can be obtained by solving the following.

$$ \mathop {\hbox{min} }\limits_{\theta \in \varTheta } \mathop \sum \limits_{i = 1}^{N} q\left( {y_{i}^{\left( t \right)} ,\theta } \right) $$

We iterate the process N times and get $\hat{\theta }^{\left( t \right)}$, which can be used for simulation [28]. An important feature of the bootstrap method is that resampling should be carried out with replacement [16, 28, 29]. In other words, this means that in the simulated data, some observations may occur more than once whereas others will not occur at all.

**Appendix 1:** Descriptive summary—data

	Women						Men				
	N	Mean	STD		Chi-2		N	Mean	STD		Chi-2
HIV testing	7,289	0.82	0.38			HIV testing	17,273	0.85	0.36		
Wealth index	7,289	2.92	1.49			Wealth index	17,273	3.31	1.41		
Education level	7,289	1.20	0.69			Education level	17,273	1.18	0.65		

	Yes		No				Yes		No		
	N	Percent	N	Percent			N	Percent	N	Percent	
HIV testing	5,995	82.25	1,294	17.750	< 0.001^a^	HIV testing	14,607	84.57	2,666	15.43	<0.001^a^
Wealth index						Wealth index					
Poorest	1,497	20.54	326	4.47		Poorest	2,114	12.24	342	1.98	
Poorer	1,053	14.45	274	3.76		Poorer	2,666	15.43	436	2.52	
Middle	1,016	13.94	248	3.40		Middle	2,722	15.76	522	3.02	
Richer	1,113	15.27	212	2.91		Richer	3,002	17.38	570	3.30	
Richest	1,316	18.05	234	3.21		Richest	4,103	23.75	796	4.61	
	5,995	82.25	1,294	17.75	< 0.001		14,607	84.57	2,666	15.43	0.011
Education level						Education level					
No education	747	10.25	131	1.80		No education	1,635	9.47	266	1.54	
Primary	3,481	47.76	846	11.61		Primary	8,961	51.88	1,740	10.07	
Secondary	1,513	20.76	300	4.12		Secondary	3,611	20.91	637	3.69	
Higher	254	3.48	17	0.23	< 0.001	Higher	400	2.32	23	0.13	< 0.001

^a^This was estimated using a one sample t-test
